# Making the business case for an addiction medicine consult service: a qualitative analysis

**DOI:** 10.1186/s12913-019-4670-4

**Published:** 2019-11-08

**Authors:** Kelsey C. Priest, Dennis McCarty

**Affiliations:** 1 School of Medicine, MD/PhD Program, Oregon Health & Science University, 3181 SW Sam Jackson Park Rd, Mail Code: L357, Portland, OR 97239 USA; 2 School of Public Health, Oregon Health & Science University-Portland State University, Portland, OR USA; 3 School of Medicine, Department of Psychiatry, Oregon Health & Science University, 3181 SW Sam Jackson Park Rd, Mail Code: L357, Portland, OR 97239 USA

**Keywords:** Opioid use disorder, Substance use disorder, Opioid agonist therapy, Buprenorphine/naloxone, Methadone, Addiction medicine consult service, Addiction consult service, Consultation service, Hospital leadership, Hospital management

## Abstract

**Background:**

As the drug poisoning crisis worsens in North America and opioid use disorder (OUD)-related hospital admissions increase, policymakers and hospital administrators are beginning to recognize the important role of hospitals in the OUD care continuum. This study explores and describes how U.S. addiction medicine physicians created and presented business propositions to hospital administrators to support the development of addiction medicine consult (AMC) services.

**Methods:**

Fifteen qualitative interviews were completed with board-certified or board-eligible addiction medicine physicians from 14 U.S. hospitals. The interviews occurred as part of a broader mixed methods study exploring hospital service delivery for patients admitted with OUD. Using a directed content analysis, the transcribed interviews were coded, analyzed, and final themes consolidated.

**Results:**

Semi-structured interviews completed with addiction medicine physicians from established (*n* = 9) and developing (*n* = 5) AMC services at 14 U.S. hospitals explored how clinical champions persuaded hospital administrators to support AMC service development. Four elements were foundational to making the “business case”: 1) describing the prevalence of substance use disorder (SUD) or OUD in the hospital; 2) identifying the negative financial impacts of not treating SUDs during hospitalization; 3) highlighting the ongoing care quality and treatment gap for hospitalized patients with SUDs; and 4) noting the success of other institutional AMC services. Study findings informed the creation of tools to support AMC service development: 1) an AMC service business case template, and 2) an AMC service design and operations resource list.

**Conclusions:**

OUD-related hospital admissions are unlikely to abate. Hospital administrators should consider innovative care delivery mechanisms to improve care for persons with OUD. AMC services may be a promising delivery mechanism to achieve this aim. For clinical and administrative champions, understanding how to communicate the potential effectiveness of this intervention to hospital leaders is an essential first step to AMC service creation.

## Background

The opioid-related overdose epidemic touches all facets of the health care delivery system, particularly increased use of acute care delivery services in emergency departments and hospitals [1]. Opioid use disorder (OUD)-related hospitalizations in the U.S. are estimated to cost $15 billion annually [2]. Research suggests that patients hospitalized with OUD may receive suboptimal care during admission [3] and upon discharge [4, 5]. These findings likely reflect the limitations in design, resources, and attention to service delivery for patients with OUD and other substance use disorder (SUDs) in the hospital.

Fortunately, effective interventions exist to treat and manage OUD and other SUDs in the hospital. Interventions for treating hospital-based OUD include: a) care delivery checklists [6–9]; b) initiating opioid agonist therapy ([OAT]—methadone and buprenorphine) [10, 11]; and c) addiction medicine consult (AMC) services [12–20]. The literature base describes the design, implementation, and effectiveness of hospital AMC services. Most published studies are single-site prospective evaluations [14, 16], retrospective assessments [18, 19], or descriptive implementation case studies [12]. Research suggests that AMC services are feasible from the perspective of the health system [20] and the patient [15], that AMC services increase the delivery of evidence-based care during hospitalization [13, 14] and upon discharge [14], and improve patient related addiction outcomes [16].

A narrative review summarized AMC service delivery attributes and the consultation process, specifically how to identify patients, the service team composition, and consultation components [21]. Little is known, however, about AMC service design and operations, beyond single-site descriptions. A qualitative analysis, based on a smaller sample of this study cohort, compared the organizational design of nine operational U.S. AMC services [17]. Generally, services in this study were staffed on the weekdays but not weekends, had interprofessional team member representation, had complex and uncertain financing, and were responsible for three practice domains: 1) hospital staff education on evidence-based SUD-related treatment; 2) delivery of psychosocial and medical services to patients with SUDs; and 3) development of hospital SUD guidance documents and policies [17]. An important facilitator leading to AMC service establishment was the creation and presentation of the service business case to hospital administrators. The strategies used by clinical leaders to make the business case for implementing AMC services are examined in this analysis.

## Methods

### Research question and theoretical underpinnings

This analysis was part of a broader mixed methods study, which asked the question: How do supply-side attributes influence hospital OAT delivery, health outcomes, and health services utilization for persons hospitalized with OUD? [22]. Supply-side attributes are the contextual elements inside and outside of a hospital that may be associated with hospital OAT delivery, such as social structures (e.g., hospital standards of care) and resources and technologies (e.g., hospital staffing, federal treatment policies) [22]. The study’s broader conceptual framework is described elsewhere [17, 22]; however, two theoretical assumptions guided this sub-analysis: the rational actor model and institutional theory. In this study, hospitals were assumed to behave as rational unitary decision-makers [23, 24] to meet the needs of their political and economic environments [25]. Institutional theory asserts that the external environment shapes organizations through a process called isomorphism which drives organizations towards homogeneity through coercion, mimicry, and normative behaviors of external stakeholders [26]. These assumptions suggest that hospital leaders choosing to implement an AMC service do so because they believe that the service is a value-maximizing activity for the hospital and that they are influenced by external policies and organizations, including other hospitals.

### Recruitment and study cohort

A publicly available list of addiction medicine fellowship programs served as the primary recruitment source [27]. Using a two-wave purposive sample technique, 45 potential key informants received email invitations to complete an interview on the topic of hospital-based services for patients with OUD and SUD. Recommendations from dissertation mentors and respondent-driven recommendations supplemented the study sample. The final, and broader dissertation study cohort included 17 key informants from 16 U.S. hospitals. The findings presented below are an analysis of the interviews from a sub-cohort (15 key informants, 14 U.S. hospitals) with established or soon to be established AMC services. The two interviews excluded from this analysis were from hospitals without an AMC service or plans to start one. Additional details on study sampling approach are available elsewhere [17, 22].

### Study tools and data collection

During the 45-to-60-min interview participants completed a short demographic survey (e.g., “What health professional degree(s) do you have?”) (see Additional file 1) and answered open-ended questions which probed for understanding of environmental and hospital supply-side attributes: “What sorts of elements within your organization supported the start of the consult service?” (see Additional file 2). Oregon Health & Science University’s Institutional Review Board reviewed and approved the study protocol and authorized the use of an information sheet rather than a formal consent process (IRB #18092). All participants gave their consent to participate in this study. The interviews were electronically recorded and transcribed. The interview guide, and demographic survey were created as tools used for a broader mixed methods study. Additional details on study tools and data collection are available elsewhere [17, 22].

### Analysis

A directed content analysis approach [28] informed the iterative transcript coding process. Qualitative analysis software (*Dedoose*) managed the analytic process [29]. An interdisciplinary review of policy, organizational behavior, systems science, economics, and health services delivery scholarship guided the development of an initial interview codebook prior to data collection [22]. The original codebook’s 5 umbrella categories, with 23 codes [22], were refined during the analytic process to reflect emergent findings [28]. The final coding scheme included eight umbrella categories and 59 codes [22]. A dual-coder process was used—after the primary investigator (KCP) completed coding, a second coder (DM) reviewed the coded transcripts and the code book. Coding discrepancies were discussed and reconciled between the two coders and the primary author consolidated final themes. Additional analytic details are available elsewhere [17, 22].

## Results

### Participant and hospital characteristics

Study participants from 14 U.S. hospitals were board-certified (*n* = 14) or board-eligible (*n* = 1) addiction medicine physicians with family medicine, internal medicine, obstetrics and gynecology, pediatrics, and psychiatry training. The mean age was 47 years and the seven women and eight men were predominantly white (*n* = 14) and Non-Hispanic or Latino (*n* = 13). Hospitals were located in the West (*n* = 4), Mid-west (*n* = 3), Northeast (*n* = 4), and South (*n* = 3) regions of the U.S. Over half of hospitals had affiliated or onsite addiction related services (e.g., opioid treatment programs [OTP] and detoxification beds). Three hospitals had OTPs and detoxification beds, two hospitals had only OTPs, three hospitals had only detoxification beds, and six hospitals had neither. Methadone and buprenorphine products for OUD treatment were on formulary at 13 of the 14 hospitals. Of the 14 hospitals in this study, nine had an AMC service and five planned to start a service. See Table 1 for a summary of hospital characteristics by region and available services.

**Table 1:** Hospital characteristics

Hospital Type	Region				Affiliated/Onsite Services			
	Midwest	Northeast	South	West	OTP	Detox	Both	Neither
Established AMC Service (*n* = 9)	3	3	1	2	1	2	2	4
Starting AMC Service (*n* = 5)	0	1	2	2	1	1	1	2
Total	3	4	3	4	2	3	3	6

### The AMC service business case

**Fig. 1:**
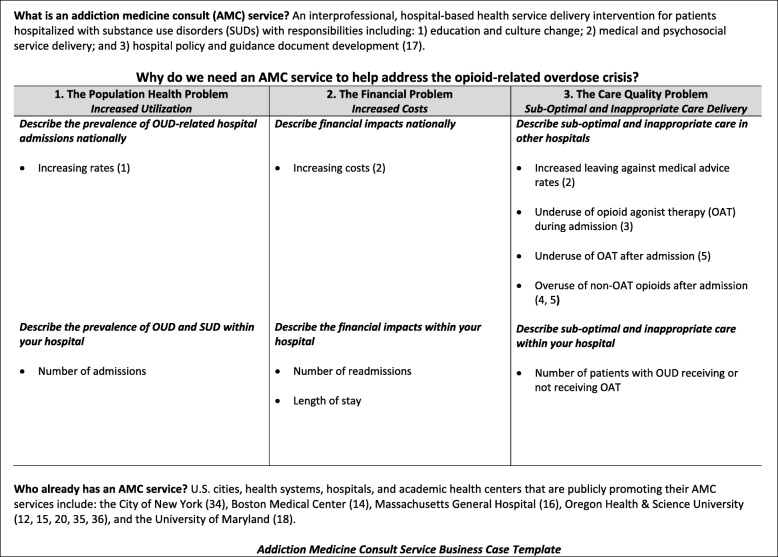
Addiction medicine consult service business case template

**Fig. 2:**
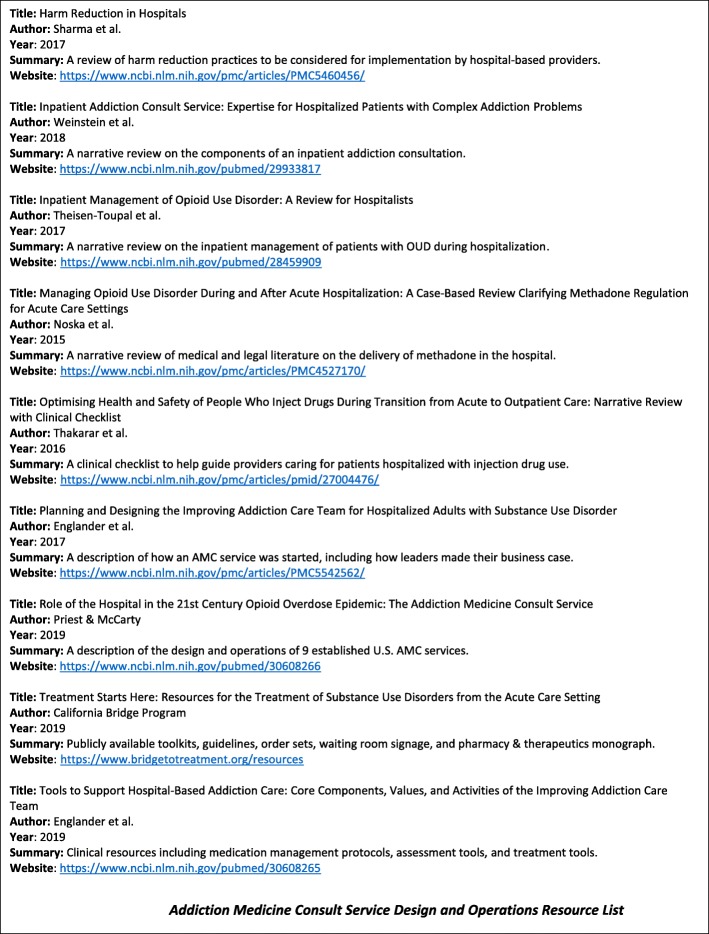
Addiction medicine consult service design and operations resource list

Hospital administrator buy-in was an essential preliminary step for AMC service establishment. The reasons administrators supported AMC services varied. Informants reported that some administrators believed that SUDs were public health and medical issues, others had clinical experience in treating SUDs, but most hospital leaders supported AMC service establishment because of the “business case.” Our analysis of the business case approach includes a description of who was involved in its creation (***The Who***), the content of the case (***The Why***), and concludes with a business case template (Fig. 1) and a resource list (Fig. 2) to support health care champions who plan to start an AMC service.

#### The Who

Addiction medicine physicians were primarily responsible for developing and presenting the AMC service business case to hospital administrators to gain their support:

Mainly me [an addiction medicine physician] knocking on a lot of doors. Putting up a fuss. And really just being a squeaky wheel.

And the reason the addiction service director, is the director, is that she was very skilled at navigating that conversation with hospital administration and convincing key hospital administrators to put up funding for this initially.

We had a few clinical champions saying ‘gee we can help these people.’ We had a clinical environment in which nursing staff and primary teams were really overwhelmed and felt they had nothing to offer patients with addiction issues. Patients were perceived as being frequently disruptive, challenging, and not easy to take care of, and people were looking for solutions.

How addiction medicine clinical leaders created these proposals varied. Two informants explained how the hospital and other organizations provided administrative support for business case development. The role of these business trained professionals was similar at both institutions, to help the clinical champion navigate the administrative challenges of launching a new hospital program by managing relationships, garnering the commitment of internal stakeholders, and organizing logistics:

I was assigned what is called a practice improvement professional who [had] a Master of Business Administration … she helped us. We started with lots and lots of meetings with stakeholders, with lots of input from people, and lots and lots of workflows, and tons of bureaucratic nightmares … Her role was frankly managing the bureaucracy, to get stakeholders on board and the hospital administration

They [hospital leadership] assigned [us] one of their [accountable care organization] interns … who helped us come up with the business case, [she] happened to be a hospitalist physician herself. That was very helpful. She was a cheerleader.

#### The Why

The specific content of the business case varied but four common elements emerged: 1) describing the increased prevalence of OUD and SUDs in the hospital or nationally; 2) highlighting the negative impact of untreated OUD and SUDs on hospital finances; 3) emphasizing the ongoing care quality and treatment gap for hospitalized patients with OUD and SUDs; and 4) noting the success of other institutions with established AMC service.

#### Elevated SUD prevalence

Addiction medicine leaders presented local or national OUD and SUD-related hospital admissions prevalence data. In one case, addiction medicine leaders used publicly available data:

It was basically just the Centers for Disease Control and Prevention data … increased opioid prescribing … increased fatality … the over dose deaths bypassing motor vehicle deaths and the newer data showing the increase in heroin across the country … we had some specific state data about prevalence of opioid addiction … so we just showed them the statistical data. Not stuff we created.

More commonly, addiction medicine champions relied on internal audits and described these data as compelling to administrators:

An audit of the inpatient [census] at our hospital 3 years ago or so found that 40% of all hospital admissions had a co-occurring substance use disorder.

Just the recognition that a lot of the hospitalized patients—between 30 and 50% of them or more have some co-occurring substance use problem...

We have such a significant proportion of patients with substance use disorders. They [hospital leadership] really felt like it was in line with the goals of the hospital and the mission to have this service available to the patients.

It is pretty much self-evident. A third or more of admissions to the hospital relate to matters pertaining to addiction.

We were able to collect data … we presented that to administration...they didn’t have the awareness of the saturation of the problem and we were really able to get buy-in from the president of the university … we basically just showed them the data...

#### Negative financial impacts

Another type of data used to narrate the need for the AMC service was how patients hospitalized with untreated SUD increased hospital resource utilization during admission and the subsequent negative impacts on hospital revenue. Clinical champions noted the importance of making the argument that the service could directly contribute to decreasing readmission penalties and could decrease lengths of stay for this patient population:

From a hospital administration standpoint there was a lot of interest in reducing length of stay and cost of care … Many of our patients had very lengthy stays.

What we ended up doing was basically through that needs assessment building a pretty strong business case around length of stay reduction and also building a business case around readmission reductions.

We made a pitch to the chief strategy officer and they agreed to support the funding … They were starting at that time to organize and create an accountable care organization … We had to pitch to them that it would be good for the accountable care organization to reduce readmissions at the same time. We also had to pitch to them that we would be revenue neutral or better in the fee-for-service system…

At one hospital, administrators were convinced to implement an AMC service because untreated patients with SUDs had longer admissions and were slowing the flow of patients from the emergency department to the inpatient wards. The informant noted that the turning point for garnering the administrators support occurred when the emergency department leadership was included in making their pitch.

#### Treatment gap and care quality issues

Addiction medicine champions explained to hospital leadership that untreated SUD in the hospital was a care quality issue. One informant described studying and presenting on patient preferences as contributing to AMC service development:

I led a needs assessment … to better understand the patient perspective … the key findings were that yes hospitalization is a reachable moment. We learned that over two-thirds of people with high risk alcohol and drug use wanted to cut back or quit and many wanted medication for addiction to start in the hospital … From a combination of firsthand experience and also from doing a fair amount of legwork talking to different stakeholder groups across the hospital, it was clear … that we didn’t have the appropriate resources in place.

Key informants frequently described the undertreatment of SUD as impacting hospital readmission rates:

Then if you looked at the 30-day readmission rate 50% of them had a co-occurring substance use disorder. There was this recognition and suggestion that untreated addiction was the driver of hospital readmission. That was one of the motivations to build the outpatient service … Rather than sending someone out with a list of phone numbers to be actually able to send them somewhere.

One of the things that brought that to their [hospital administrators] attention was the issue of readmission. There was a pretty high readmission rate among patients with SUD diagnoses. And, that under the current reimbursement system hospitals are penalized for that amount. So that drew their attention to this issue.

#### Success of other institutions

Addiction medicine champions strategically leveraged the success of other institutions already providing inpatient resources for SUD treatment to convince administrators of the value of the service:

In addition to the data that we had collected, it was honestly some healthy competition. I was saying look [institution A] has one of these, [institution B] has one of these, [institution C] has one of these. New York City is creating a city-wide service through the health and hospitals program. It is really silly that we don’t have one. That caught their ear and they were able to look at the data a little more …

The previous university I worked for had an addiction medicine fellowship and they closed it down due to preference from the administration … We were able to basically show [our hospital leadership] that we don’t have this in our state anymore. Our university could be the driving force for this.

## Discussion

Historically, hospital administrators, national hospital associations, and the Joint Commission have provided limited attention and resources to the care of hospitalized patients with addictive disorders, especially those with OUD. However, in the midst of the opioid-related overdose crisis, attention to treatment in the hospital is growing [30]. National and state policymakers are beginning to address care deficits in this setting. The Centers for Medicare & Medicaid Services (CMS) now require the use of the American Society of Addiction Medicine (ASAM) levels of care for state Medicaid programs applying for §1115 waivers to redesign SUD delivery systems [31] and hospitals are a part of this care continuum [32]. Further, recent legislation in Massachusetts requires emergency department clinicians to offer and provide OAT to patients seeking care with an OUD [33]. This is a policy that could be readily extended to care delivery in the inpatient setting.

Unfortunately, interest in improving hospital care for patients with OUD and other SUDs, by and large, has not been driven by ethical, moral, and legal arguments. Our informants noted that these arguments were not sufficient for convincing most high-level hospital administrators to implement an AMC service. Garnering the support of high-level hospital leadership instead relied primarily on articulating how the service aligned with hospital goals and how the service could operate as a financially value-maximizing activity; thus, clinical champions pitched the service as a business proposition. How this information was gathered and subsequently packaged for presentation depended on the experience, expertise, and training of the addiction medicine physicians and available resources at each hospital.

The prevalence of U.S. AMC services is unknown and a centralized list or repository of service locations has yet to be created. There are several U.S. cities, health systems, hospitals, and academic health centers publicly promoting and publishing on the existence of their respective AMC services. In New York City, New York Health and Hospitals launched a city-wide program to implement six AMC services at six hospitals—the Consult for Addiction Treatment and Care in Hospitals (CATCH) program. The CATCH program has an evaluation plan in place and will be the first multi-site study on the effectiveness of AMC services [34]. Other institutions with public-facing programs include: Boston Medical Center [14], Massachusetts General Hospital [16], Oregon Health & Science University [12, 15, 20, 35, 36], and the University of Maryland [18].

At least two groups have circulated tools designed to improve care for patients with OUD and SUDs in the hospital setting—the California Bridge Program and the Improving Addiction Care Team (IMPACT) at Oregon Health & Science University. The California Bridge Program, affiliated with the Public Health Institute, provides open-source resources related to the care of patients with OUD and SUD in the hospital setting, including but not limited to: inpatient guidelines, order sets, patient materials, pharmacy and therapeutics committee materials, and OAT financing and billing resources [37]. The IMPACT team recently published a compendium of resources including medication management protocols (e.g., withdrawal protocol), assessment tools (e.g., social work SUD assessment), treatment tools (e.g., patient safety care plan), and other resources (e.g., sample letter to judge or parole officer) [35]. To date, neither group has published tools on how to make the AMC service business case to hospital administrators; thus, it is the synthesis of the findings from this study, paired with prior literature review [22], that informed the development of two tools to fill this gap: 1) an AMC service business case template (Fig. 1); and 2) an AMC service design and operations resource list (Fig. 2).

The purpose of Fig. 1 is to provide evidence and rationale to convince hospital administrators why an AMC service would benefit their respective hospital. Figure 1 includes a description of what an AMC service is, why a service should be created to help address the opioid-related overdose epidemic, and which organizations are national leaders of this care delivery intervention. Figure 2 is a list and summary of recently published resources related to AMC service design and operations to support clinical champions planning to launch a service.

The primary study limitation is transferability, because most hospitals in this sample were affiliated with urban academic health centers and had above average access to addiction-related resources (e.g., education, staff, research). The findings may be less applicable to hospitals without addiction medicine experts, addiction medicine trainees, or that exist in lower-resourced settings. Another study limitation was the heterogeneity of the involvement of the study key informants in AMC service establishment and operations. Differences in positionality, observer versus implementer, may influence the perceptions of the informant [38].

## Conclusion

As OUD-related hospitalizations increase, and the drug poisoning crisis worsens, hospital administrators should look to innovative care delivery mechanisms to improve care and outcomes for patients with OUD. The AMC service may be a service delivery intervention to achieve these aims. For clinical and administrative champions, understanding how to communicate the relevance and potential effectiveness of this organizational intervention, in the midst of the opioid overdose epidemic, to hospital leaders is a foundational first step to improving care in this setting for patients hospitalized with OUD.

## Supplementary information

**Additional file 1.** Key Informant Demographic Survey

**Additional file 2.** Semi-Structured Interview Guide

## Data Availability

Not applicable.

## Abbreviations

AMC: Addiction medicine consult service
ASAM: American Society of Addiction Medicine
CATCH: Consult for Addiction Treatment and Care in Hospitals
CMS: Centers for Medicare & Medicaid Services
IMPACT: Improving Addiction Care Team
OAT: Opioid agonist therapy
OUD: Opioid use disorder
SUD: Substance use disorder
