# Supplementary material for: Making the business case for an addiction medicine consult service: a qualitative analysis
Source: BMC Health Serv Res. 2019 Nov 8;19:822. doi: 10.1186/s12913-019-4670-4 (PMC6842195; doi:10.1186/s12913-019-4670-4)
Supplement: Supplementary file 1 — Additional file 1. Key Informant Demographic Survey [file 12913_2019_4670_MOESM1_ESM.docx]

**Supplement 1**

**Key Informant Demographic Survey**

What is your age?

- <25
- 25-29
- 30-34
- 35-39
- 40-44
- 45-49
- 50-54
- 55-59
- 60-64
- 65-69
- 70 or older

With what gender do you identity?

- Woman
- Man
- Other __
- Decline to Answer

How do you racially identify?

- American Indian/Alaska Native
- Asian
- Native Hawaiian or Other Pacific Islander
- Black or African American
- White
- More than one race
- Decline to Answer

What ethnicity best describes you? [pick one]

- Hispanic or Latino
- Not Hispanic or Latino
- Decline to Answer

What health professional degree(s) do you have? And what year did you graduate from each program(s)?

Which profession best describes your current work?

- Hospital administrator/manager
- Nurse
- Pharmacist
- Physician
  - Specialty:
  - Addiction Medicine Board Certification? (y/n)
- Social worker
- Behavioral health specialist
- Legal counsel
- Other___

For how many years have you worked at your respective hospital?

- <1
- 1-2
- 3-5
- more than 5

For how many years have you been the fellowship program director?

- <1
- 1-2
- 3-5
- more than 5

For how many years have you worked on the addiction consult service (if applicable)?

- <1
- 1-2
- 3-5
- more than 5

Does your hospital have methadone on the formulary for treatment of OUD?

- Yes
- No
- Unknown

Does your hospital have buprenorphine on the formulary for treatment of OUD?

- Yes
- No
- Unknown

*From: Priest KC. Hospital-based services for patients with opioid use disorder: A study of supply-side attributes. Dissertations and Theses. 2019;Paper 4829. 10.15760/etd.6705.*
