# Supplementary material for: Making the business case for an addiction medicine consult service: a qualitative analysis
Source: BMC Health Serv Res. 2019 Nov 8;19:822. doi: 10.1186/s12913-019-4670-4 (PMC6842195; doi:10.1186/s12913-019-4670-4)
Supplement: Supplementary file 2 — Additional file 2. Semi-Structured Interview Guide [file 12913_2019_4670_MOESM2_ESM.docx]

**Supplement 2**

**Semi-Structured Interview Guide**

**ID #: Date:**

**Introduction**

My name is Kelsey Priest, and I am a fourth year MD/PhD student at Oregon Health & Science University (OHSU) and a doctoral student at OHSU-Portland State University School of Public Health in the Health Systems and Policy program. As part of the requirements of the doctoral program, I am conducting dissertation research to study the treatment of persons with opioid use disorder (OUD) in the hospital setting. As the [position of research participant] for the [hospital name], you have been identified as knowledgeable about the treatment of persons with opioid use disorder at [hospital name].

This project is an opportunity to learn more about the health care service patterns of opioid agonist therapy (OAT) delivery in the hospital setting for persons with opioid use disorder. The purpose of this interview is to capture your perspectives about your organization’s hospital opioid use disorder care delivery policies, procedures, and practice, specifically:

1. Hospital OUD care delivery policies, procedures, and practice.
2. Barriers and facilitators to hospital OUD treatment and OAT delivery.
3. Ideas or solutions for enhancing hospital OUD treatment and OAT delivery.

The interview will last between 45 and 60 minutes, depending on the length of your answers. If you describe any hospital-based policies during the interview I will follow-up via email after the interview with a request to see them with your permission and I may also follow up with additional clarifying questions.

**Consent**

I have provided the consent form ahead of time, and I want to ensure that you are clear on the expectations of participation. I assume that your presence here today indicates you have read the consent form. Do you have any questions about your participation in this research study? I would now like to receive verbal consent to participate from you.

*<Obtain verbal consent>*

**Audio Recording Instructions**

With your permission, I will take notes and record the interview. Your participation in this interview is voluntary; you do not have to answer any question that you do not want to answer and you may stop the interview at any time. All individual responses will be kept confidential.

The recording and my notes will help me to accurately represent our discussion; no one else will ever hear the recordings or see the written transcripts. If there are things that you tell me that you do not wish repeated, please indicate this so that I do not include those comments in any summaries or reports that I develop from this interview.

Similarly, if at any time you would like me to stop recording, please indicate this and I will turn off the recorder. Findings will be reported in the aggregate with larger themes identified within and across hospitals. Quotes will be selected to illustrate these broader themes and will be presented without attribution to individuals. Do I have your permission to record this interview?

<*Obtain verbal consent, and turn on the recording device>*

At this time, do you have any other questions or concerns?

*<After addressing any questions and/or concerns>*

Then let us begin.

**Questions**

1. We will start with a brief demographic survey.

*<Reads questions from key informant demographic survey>*

1. **Role and Introductions.** Please tell me briefly about your role at your institution and within the hospital.
2. **Responsibility of Care.** Who (what service) within the hospital is responsible for the clinical management of an OUD during hospitalization?
   1. Probe: Dependent on clinical scenario (e.g., surgical vs. non-surgical or acuity).
3. **Care Delivery Mechanisms.** Does your hospital have an addiction consult service?
   1. If yes, when did the service start? What was the context or catalyst for this change?
      1. Probe: Since that time, what shifts in culture and/or stigma of persons with OUD or other addictive disorders have occurred?
      2. Probe: How have hospital policies, procedures, or guidelines changed over time related to care for persons with OUD since the establishment of the service?
      3. Probe: What were the facilitators for starting the service?
         1. Probe: Within the organization?
         2. Probe: Outside the organization?
      4. Probe: What were the barriers to starting the service?
         1. Probe: Within the organization?
         2. Probe: Outside the organization?
   2. If no, is this something that is being considered? If not, why do you think that is?
4. **Addiction Consult Service Structure and Design.** What is the current design of the addiction consult service?
   1. Probe: Describe the team composition (e.g., what professionals are on the team).
   2. Probe: Describe the services provided by the consult service (e.g., medication management, pain consultation, harm reduction).
   3. Probe: Describe the availability of the service (e.g., weekdays only, 24-7 coverage).
5. **Current Policies.** What are your hospital’s current policies and procedures (e.g., guidelines) for OUD management and/or withdrawal?
   1. Probe: Specifically, does your hospital have policies or procedures on the continuation of methadone or buprenorphine for OUD treatment during hospitalization?
      1. If so, please describe the policies.
      2. Have these changed over time? If yes, how so?
   2. Probe: Does your hospital have any policies or procedures on the induction of methadone or buprenorphine during hospitalization?
      1. If so, please describe the policies
      2. Have these changed over time? If yes, how so?
   3. Probe: Does your hospital have any policies or procedures on the use of buprenorphine or methadone to manage withdrawal?
      1. If so, please describe the policies.
      2. Have these changed over time? If yes, how so?
   4. Probe: If applicable, do these policies or procedures reflect practice generally?
6. **Organizational Barriers.** What do you think are some of the organizational barriers to implementing policies and procedures for caring for persons with OUD at your hospital?
   1. Probe: Physical environment/location?
   2. Probe: Leadership?
   3. Probe: Resources and staffing?
   4. Probe: Policy implementation process?
   5. Probe: Hospital culture and understanding of OUD?
7. **External Barriers.** What do you think are some of the external barriers to implementing policies and procedures for caring for persons with OUD at your hospital?
   1. Probe: Federal or local policies? (e.g., federal regulations or insurance regulations)
   2. Probe: System service delivery issues (e.g., care transitions)?
   3. Probe: Other local hospitals?
8. **Organizational Facilitators**. What do you think are some of the organizational facilitators for implementing policies and procedures for caring for persons with opioid use disorder at your hospital?
   1. Probe: Physical environment/location?
   2. Probe: Leadership?
   3. Probe: Resources and staffing?
   4. Probe: Policy implementation process?
   5. Probe: Hospital culture and understanding of OUD?
9. **External Facilitators.** What do you think are some of the external facilitators for implementing policies and procedures for caring for persons with opioid use disorder at your hospital?
   1. Probe: Federal or local policies?
   2. Probe: System?
   3. Probe: Other local hospitals?
10. Is there anything else that we have not discussed about the treatment of OUD in your hospital?

**Closing the Interview**

Thank you for participating in this interview, and for your thoughtful comments, insights and candor. I am meeting with approximately 19 other key informants from other hospitals across the United States. I will be analyzing and synthesizing the key themes and issues that emerge over the course of the study. If you think of anything else, please contact me. Findings from the interviews will be included in my dissertation and read by the OHSU-PSU School of Public Health faculty serving on my dissertation committee. At the conclusion of the study, I would be happy to share a report of the aggregated findings with you. May I contact you if I have any follow up questions? Again, thank you for your time and willingness to participate.

*From: Priest KC. Hospital-based services for patients with opioid use disorder: A study of supply-side attributes. Dissertations and Theses. 2019;Paper 4829. 10.15760/etd.6705.*
